# Development of Embodied Sense of Self Scale (ESSS): Exploring Everyday Experiences Induced by Anomalous Self-Representation

**DOI:** 10.3389/fpsyg.2016.01005

**Published:** 2016-07-05

**Authors:** Tomohisa Asai, Noriaki Kanayama, Shu Imaizumi, Shinichi Koyama, Seiji Kaganoi

**Affiliations:** ^1^Nippon Telegraph and Telephone Communication Science Laboratories, Human Information Science LaboratoryKanagawa, Japan; ^2^Department of Psychiatry and Neurosciences, Institute of Biomedical and Health Sciences, Hiroshima UniversityHiroshima, Japan; ^3^Graduate School of Engineering, Chiba UniversityChiba, Japan; ^4^Department of Rehabilitation, Geisei HospitalKochi, Japan

**Keywords:** sense of self, embodiment, agency, ownership, narrative

## Abstract

The scientific exploration of the self has progressed, with much attention focused on the Embodied Sense of Self (ESS). Empirical studies have suggested the mechanisms for self-representation. On the other hand, less attention has been paid to the subjectivity itself of the self. With reference to previous studies, the current study collected items that reflect the ESS and statistically extracted three factors for it: *Ownership*, *Agency*, and *Narrative*. The developed questionnaire [Embodied Sense of Self Scale (ESSS)] showed good enough validity and reliability for practical use. Furthermore, ESSS discriminated schizophrenia, a disorder of the ESS, from controls. We discuss the factorial structure of ESS and the relationship among factors on the basis of the current results.

## Introduction

One of the distinctive scientific interests in this decade has been characterized by studies of the self. Recent psychological and neuroscience studies have focused on examining the self-representation in the brain. This trend started with revisiting our sense of body and action (Gallagher, 2000; Jeannerod, 2007; Blanke et al., 2015). Since these senses can be examined empirically, how the subjective feeling of “my own body” or “my own action” is elicited has been revealed in behavioral, neural, and computational approaches (Miall and Wolpert, 1996; Wolpert, 1997; Blakemore et al., 2002; Frith, 2005; Tsakiris et al., 2007; Blanke et al., 2015) and also applied to induce virtual presence and immersion in Cyberpsychology in tandem with recent technological progress (see for review, Slater et al., 2008; Blanke et al., 2015). The sense of body (e.g., “this is my own hand”) needs multimodal sensory integration (Kilteni et al., 2015), while the sense of action (e.g., “I myself am moving the hand”) derives from matching motor predictions with actual feedback or outcomes (David et al., 2008). These subjective feelings of the self, which are grounded within our own body or sensorimotor system (Weiss et al., 2014), might be called as the Embodied Sense of Self (ESS).

Though this progress based on empirical or objective measures has revealed mechanisms for ESS, its subjectivity itself has been treated as secondary. As a result, the phenomenological aspects of the ESS have not yet been fully examined (e.g., Longo et al., 2008; Haggard and Chambon, 2012): this study, entitled as “What is embodiment?”, specifically focused on the phenomenological sensations in the rubber hand illusion (RHI; Botvinick and Cohen, 1998; see also Discussion). A difficulty in exploring the subjective ESS is that we are not always aware of the feeling of self (i.e., pre-reflective self, Gallagher, 2000; Legrand, 2007; Praetorius, 2009; Trehub, 2009) because the brain automatically regulates many functions including body and action unless an operational error is detected (Nielsen, 1963; Fourneret and Jeannerod, 1998; Knoblich and Kircher, 2004; Tajadura-Jimenez et al., 2012; Asai, 2015b). Therefore, a possible approach for the awareness for the self would be to focus on those errors, namely, everyday experiences that might be caused by an abnormal ESS (e.g., Sivertsen and Normann, 2015). That was how the current study explored the factorial structure of the ESS, and as a result, and more importantly, the current study developed a questionnaire that reflects on that factorial structure. The questionnaire could be a useful tool for measuring an anomalous ESS, especially for patients with mental illnesses, such as schizophrenia. Similar approaches based on subjective reports for the sense of self have already been reported (e.g., Jones et al., 2008; DaSilveira et al., 2015). For example, prototype scales for the sense of action (i.e., “sense of agency”, see below) have been developed (Asai et al., 2009b; Polito et al., 2013). Parnas et al. (2005a,b) have suggested a checklist for an abnormal sense of body and action (i.e., “minimal self”, see below). With reference to these previous studies, the current study aimed to develop a scale for a more comprehensive ESS and examine its factorial structure.

What factors can we assume contribute to the ESS? First of all, as noted above, the sense of body, the physical boundary between the self and the environment, is fundamental for the ESS. Once we have a body, we can move it (Asai, 2015a). The body enables us to act as an agent in the world. Gallagher (2000) has theorized this online sensory level of self as the *minimal self*, a self devoid of temporal extension, wherein the “sense of ownership” means the feeling of owning my own body and, therefore, the experiencing of sensations on it (Gallagher, 2000; Tsakiris, 2010), while the “sense of agency” means the feeling of causing my own action (Haggard and Chambon, 2012). This concept has been extended by empirical evidence so that the sense of ownership includes not only my own body but external tools (Iriki et al., 1996; Maravita and Iriki, 2004), and the sense of agency also includes the sense of self-attribution of an action’s outcome (i.e., sensory event) in the external world (Haggard, 2005; Sato and Yasuda, 2005). In this sense, “I” seems to be the recursive feeling both as a “physical existence” that can be owned and as an “intentional agent” that can affect the world.

The minimal self, however, might be imperfect for self-representation. Gallagher (2000) also referred to the *narrative self*, the self with temporal extension and continuity across time (White, 2015). Right after we have a sensory experience, it goes past. As the sensory experiences that construct minimal self accumulate across time as memory (i.e., autobiographical source memory, Sugimori et al., 2012; Sugimori and Asai, 2015), what we have done and what we have experienced construct a narrative for the self (Habermas and Kober, 2015). This temporal extension of selfness might generally be referred to as identity or personality (Ramachandran, 1998; Damasio, 1999; Habermas and Kober, 2015) so that we can imagine what we will do in the future and simulate how we will behave in a certain situation on the basis of past experiences. At this stage, the self finally achieves a uniqueness (i.e., identity) that includes behavioral traits and prospective decision making patterns (e.g., prospective agency, Chambon and Haggard, 2012; Chambon et al., 2015). In this sense, the narrative self simply includes autobiographical memory and identity (Gallagher, 2000). The self, not a simple substance (e.g., minimal self) but something that is maintained by mental experience (e.g., personal identity), is not an already existing entity that produces the narrative. Instead, it is the “product of the narrative itself” (Di Francesco, 2008).

According to these discussions, the ESS seems to include the minimal self and narrative self: the former further contains the sense of ownership and agency, while the latter contains autobiographical memory and identity. Though the relationship between minimal and narrative self is still unclear (Gallagher, 2000), these two domains might construct a unified self-representation in terms of embodiment (Damasio, 1999). The mental experience involving the minimal and even narrative self is not simply cognitive, but also emotional and embodied (Gallagher, 2004), where we refer to ourselves as an identical or narrative entity through our own online sensory experiences in body and action and also through its memorized experiences (see also Discussion). Ramachandran (1998) also suggested similar essential factors for self-representation: *embodied self* (=ownership), *executive self* (=agency), *mnemonic self* (=autobiographical memory), and *unified self* (=identity). The current study refers to these potential factors as ownership, agency, continuity, and uniformity. We first collected items in accordance with these factors with reference to previous studies and then developed a questionnaire for the ESS with a statistical procedure to confirm its validity and reliability by administering five successive surveys (the total number of participants were 1167). Specifically, Survey A explored the factorial structure of ESS: the potential factors including *ownership* and *agency* for minimal self as well as *continuity* and *uniformity* for narrative self were hypothesized to comprise the ESS. Survey B confirmed the validity of the developed questionnaire [the Embodied Sense of Self Scale (ESSS)] by examining correlations with other to-be-correlated scales, including the schizotypal personality scale. Survey C further examined the relationship between the ESSS and potentially related scales, including self-esteem, and self-efficacy questionnaires. Survey D confirmed the reliability of the ESSS in terms of temporal consistency. Survey E examined differences in the ESSS among schizophrenics and amputees. We discuss the factorial structure of the ESS on the basis of that developed ESSS questionnaire and the statistical results.

## Materials and Methods

The questionnaires used in the current study, including our newly developed one here, were in Japanese for Japanese participants. We used Japanese versions of the existing questionnaires shown below. All of them have been reported to have good validity and reliability. The items in the newly developed one are expressed in English here with back-translation.

### Item Collection

With reference to previous studies, we collected and created 120 potential items that reflect the ESS (Supplementary Material S1). These studies include the following questionnaire studies: the schizotypal experience scales (linked with ESS; Launay and Slade, 1981; Claridge and Broks, 1984; Bentall et al., 1989; Raine, 1991; Mason et al., 1995; Waters et al., 2003; Cyhlarova and Claridge, 2005; Sugimori et al., 2009), depersonalization scales (linked with ownership; Putnam, 1997; Sierra and Berrios, 2000), and agency or minimal self scales (Parnas et al., 2005b ; Asai et al., 2009b). We also considered many other empirical, theoretical, and phenomenological studies to prepare for potential new items (e.g., Cahill, 1996; Iriki et al., 1996; Daprati et al., 1997; Botvinick and Cohen, 1998; Blakemore et al., 2000; Frith et al., 2000a; Peled et al., 2000; Fourneret et al., 2001; Franck et al., 2001; Platek and Gallup, 2002; Wegner, 2003; Blanke, 2004; Knoblich and Kircher, 2004; Maravita and Iriki, 2004; Sato and Yasuda, 2005; Asai and Tanno, 2007, 2008, 2013; Jones and Fernyhough, 2007; Asai et al., 2008, 2009a, 2011; David et al., 2008; Longo et al., 2008; Johns et al., 2010; Newport et al., 2010; Hauser et al., 2011; Hommes et al., 2011; Sugimori et al., 2011a,b, 2012; Sugimori and Asai, 2015). *Ownership* was assumed to include items like “Sometimes it feels like my body is jerky like a robot.” *Agency* might include “I sometimes bump into things or people when I am out walking.” *Continuity* might include “I cannot remember what I did during that period because my memory was fuzzy.” *Uniformity* might include “Sometimes I feel that I no longer know my own personality.” A tense and an expression for items were unified so that a higher score on a five-point Likert scale means a more anomalous ESS. This 120-item temporal scale is called the Embodied Sense of Self Scale temporal (ESSSt). The instruction was “Please indicate the extent to which the following statements generally apply to you by circling the corresponding number (1–5) next to the statement. (i) Strongly disagree, (ii) Disagree somewhat, (iii) Neither disagree nor agree, (iv) Agree somewhat, and (v) Strongly agree”.

### Participants and Procedure

#### Survey A

Japanese university students (*N* = 718, male = 364, average age = 19.7, *SD* = 2.60) participated in this survey during psychology classes where the 120-item ESSSt was conducted so that the items would be selected with a statistical procedure. Then we explored the factorial structure. At this stage, we finally developed a 25-item ESSS that was used in the following surveys (see the “Results” Section for details).

#### Survey B

A community sample was recruited individually (*N* = 106, male = 72, average age = 30.7, and *SD* = 6.67). They answered some other questionnaires for confirming criterion-related validity as well as the ESSS. The prototype agency scale [Sense of Agency Scale (SOAS); Asai et al., 2009b] and multidimensional schizotypy scale [Oxford–Liverpool Inventory of Feelings and Experiences (O-LIFE); Mason et al., 1995] were used for convergent validity, while the empathy scale [Interpersonal Reactivity Index (IRI); Davis, 1980, 1983] was used for divergent validity. It was necessary to discriminate between the sense of self (targeted by the current scale) and the sense of others (e.g., empathy) since they might somehow be related to each other (Miall, 2003; Sinigaglia and Rizzolatti, 2011). The IRI, one of the commonly used scales for empathy, might simply correlate with the ESSS in a weak manner, but we expected that this superficial correlation would diminish after controlling for other potentially mediating variables like schizotypy (e.g., Asai et al., 2011).

#### Survey C

We asked a marketing company to conduct a web survey in order to collect an area-independent community sample (*N* = 153, male = 74, average age = 26.0, and *SD* = 2.74). They answered the self-esteem scale [Rosenberg Self Esteem Scale (RSES); Rosenberg, 1965] and self-efficacy scale (Generalized Self Efficacy Scale, GSES, Sherer et al., 1982) as well as the ESSS for confirming criterion-related validity again from a more explorative perspective. Self-esteem and self-efficacy have a concept similar to (but not totally the same as) the ESS. We, therefore, hypothesized a weak significant correlation between the ESSS and RSES or GSES (convergent validity).

#### Survey D

The web survey was conducted again for the same sample who answered in Survey C after approximately a month interval (*N* = 132, male = 62, average age = 25.7, and *SD* = 3.31). In order to confirm re-test reliability, they answered the ESSS again.

#### Survey E

The final survey was to confirm validity of the ESSS in terms of a clinical perspective. Schizophrenic patients (*N* = 15, male = 9, average age = 60.9, and *SD* = 6.45, average year of hospitalization = 10.7, average medication = 657.8 CP-mg) and upper limb amputees (*N* = 11, male = 11, average age = 67.1, and *SD* = 12.1, average of years since amputation = 43.3) as well as age-matched healthy controls (*N* = 32, male = 25, average age = 66.3, and *SD* = 8.00) answered the ESSS. The schizophrenic patients, none of whom had a history of cerebrovascular diseases, were diagnosed as chronic schizophrenics on the basis of DSM-V (American Psychiatric Association, 2013) and were clinically stable at the time of the survey. There was no statistical difference in age among three groups [*F*(2,55) = 2.42, *p* = 0.10]. We hypothesized that people with schizophrenia have an anomalous ESS (enhanced score in the ESSS), indicating convergent validity, compared with healthy controls and even amputees, indicating divergent validity. This is because schizophrenia might be a disorder of the self, characterized by a disturbed self-representation in the brain (Frith et al., 2000b; Cermolacce et al., 2007; Jeannerod, 2009). Such a disturbed self-representation has not been suggested for amputees since they have no self-related mental symptoms like hallucinations or delusions, though both schizophrenics and amputees claim an anomalous feeling or inconvenience in their bodily sensations and motor abilities (Walker, 1994; Meier, 2001; Putzhammer and Klein, 2006; Mayer et al., 2008; Gallese and Ferri, 2014; Imaizumi et al., 2014).

### Ethical Treatment

The procedure and policy in treating personal information in the current surveys were approved by the local ethics committees of each responsible institution (Chiba University, Geisei Hospital, and NTT Communication Laboratories).

## Results

The data obtained in the current surveys were analyzed by SPSS 17.0j as follows.

### Item Selection and Factor Analysis (Survey A)

The 120-item ESSSt was answered by 718 university students. First of all, 40 items were excluded because their distributions were skewed, with their average scores ± 1 *SD* out of the scoring range (1–5), indicating ceiling or floor effects. At this stage, the extreme items like “I sometimes feel as if my body doesn’t actually exist.”, “Sometimes I feel like I am in a dream state where I hallucinate and have weird experiences.” or “When I am really tired, I get motion sickness when I am walking.” were dropped. Though these items might be a direct expression of an anomalous ESS, statistically speaking, these experiences are so rare in the general population on a daily basis that they should not be included in the following analysis. The first explorative factor analysis was conducted for the remaining 80 items. Because the assumed factors were thought to be correlated with each other, promax rotation with maximum likelihood method was applied. Since there were four assumed factors (ownership, agency, continuity, and uniformity), the four factor model was applied (the number of factors was fixed at four). However, the potential items for continuity and uniformity were not separable for each factor, while the items for ownership and agency were. Therefore, the items for continuity and uniformity were assumed again to be loaded to one factor: *Narrative*. At this stage, items with smaller loads in the above factor analysis (<0.3), items that had loads to multiple factors, and items for specific people (e.g., regarding driving, cellphones, or PC) were removed (for example, “It sometimes seems like the cellphone in my pocket or bag is vibrating.” is not applicable for non-cellphone users). After that, the second confirmative factor analysis with a three-factor model (ownership, agency, and narrative) was conducted for the remaining 40 items, which revealed three interpretable factors as we hypothesized. After items with smaller loads in this factor analysis (<0.3) and items that had loads to multiple factors were removed again, the final factor analysis reconfirmed the three factors of the 25-item ESSS (eigenvalues were 5.46, 2.00, 1.51, 1.09, and 1.03 for factor 1, 2, 3, 4, and 5, respectively; **Table 1**). Though the item “I think I am more ticklish than other people.” had a smaller load (0.30) than the others (>0.35), the fact that this item was included in *Agency* should be important in terms of the factorial validity of the ESSS. Therefore, this item was not removed (see below for the interpretation of *Agency* factor).

**Table 1:** The items and the factorial structure of ESSS.

	Item	Ownership	Narrative	Agency	Communality
1	Sometimes the clothes I am wearing feel heavy	0.652	-0.073	0.035	0.390
2	Sometimes it feels like my body is jerky like a robot	0.637	-0.082	0.053	0.374
3	Thoughts that come to mind seem to be someone else’s	0.529	0.116	-0.006	0.365
4	I am strangely bothered by the way clothing rubs against my skin	0.430	-0.063	0.137	0.214
5	When out walking, I rarely notice my reflection in mirrors or shop windows	0.429	-0.047	-0.116	0.141
6	Sometimes I become aware that my body has become chilled	0.423	0.090	0.024	0.245
7	Sometimes my existence seems to lack a sense of reality	0.404	0.294	-0.127	0.330
8	Sometimes I sense that my body is very light	0.387	-0.109	0.038	0.120
9	When I am doing something, it seems like I am observing myself from a distance	0.367	0.187	-0.010	0.248
10	Sometimes I feel that I no longer know my own personality	0.025	0.665	-0.025	0.446
11	My personality changes depending on the setting and the situation	-0.043	0.599	-0.109	0.279
12	No matter how hard I concentrate, unrelated thoughts intrude upon my thinking	-0.159	0.555	0.179	0.337
13	When a song gets stuck in my head, it is really hard to turn it off	-0.059	0.541	0.070	0.298
14	It seems like the person I was in the past and the person I am today are completely different	0.126	0.449	-0.020	0.275
15	I feel like sometimes people misunderstand my personality	0.160	0.427	-0.099	0.245
16	I cannot remember what I did during that period because my memory was fuzzy	0.089	0.397	0.111	0.273
17	I sometime recall things that make me smile to myself	-0.141	0.379	0.140	0.157
18	I lose things without even being aware they are lost	-0.020	-0.041	0.633	0.368
19	I tend to drop things when I carry things around	0.147	-0.051	0.628	0.452
20	I sometimes bump into things or people when I am out walking	0.035	0.102	0.534	0.372
21	I have been told that my voice is too loud	-0.089	-0.094	0.491	0.186
22	Something may attach itself to me without me realizing it	-0.004	0.062	0.439	0.223
23	It is difficult to grope around for something without being able to see it	0.013	0.048	0.368	0.160
24	Sometimes I forget what I was going to say	-0.043	0.184	0.367	0.218
25	I think I am more ticklish than other people	0.063	-0.006	0.303	0.109

	Inter-correlation among factors (after rotation)	1	2	3	

		–	0.605	0.402	
			–	0.512	
				–	

*These items in English were back-translated from the original Japanese items that were used in the current study.*

The first factor, *Ownership*, includes nine items like “Sometimes the clothes I am wearing feel heavy.”, “Sometimes I become aware that my body has become chilled.” and “When I am doing something, it seems like I am observing myself from a distance.” This factor reflects the sense of ownership over one’s own body and property (e.g., external operational tools), and further on the sense of the existence or reality of ourselves. The recent updated understanding of ownership is included in these items, where ownership is not only for our own body and its counterpart but for even selfness, whose disorder could cause depersonalization (Putnam, 1997) (see also the “Discussion” Section).

The second factor, *Narrative*, includes eight items like “My personality changes depending on the setting and the situation.”, “No matter how hard I concentrate, unrelated thoughts intrude upon my thinking.”, and “It seems like the person I was in the past and the person I am today are completely different.” This factor reflects the continuity (i.e., temporal extension through autobiographical memory) and uniformity (i.e., uniqueness known as identity, personality, and behavioral traits) of the self. Though these two aspects of the “narrative self” had been assumed to have their own factor, statistical results indicated they contributed to just one factor simultaneously. Compared with the first and the third factor shown below, there is still much to be clarified about the narrative self (Gallagher, 2000) (see the results of “Survey C” and “Discussion”).

The third factor, *Agency*, includes eight items like “I tend to drop things when I carry things around.”, “It is difficult to grope around for something without being able to see it.”, and “Sometimes I forget what I was going to say.” This factor reflects the sense of controllability of a target, including our own body, and the intentionality of our own action. In particular, though the item “I think I am more ticklish than other people.” might be superficially unrelated to agency, self-ticklishness (i.e., sensory attenuation, Blakemore et al., 1998) is thought to be an implicit measure for agency according to the computational theory of motor control (Wolpert, 1997). Therefore, the fact that not the factor *Ownership*, that is related to bodily sensation, but the factor *Agency* included this item supports the factorial validity of the ESSS.

The descriptive statistics of the current surveys (Surveys A–D) are summarized in **Table 2**. Since there was no statistical gender difference in the ESSS total score in Survey A, the following analysis excluded gender difference.

**Table 2:** Descriptive statistics of the current study.

	Survey (*N*)	Average (*SD*)	Minimum–Maximum
ESSS	Survey A (718)	73.7 (13.8)	33–118
Ownership		20.3 (6.23)	9–43
Narrative		28.1 (5.68)	8–40
Agency		25.1 (5.70)	8–40
O-LIFE	Survey B (106)	40.5 (11.7)	14–70
Positive		6.97 (5.41)	0–24
Negative		13.3 (1.89)	9–17
Disorganized		12.3 (5.14)	0–24
Impulsive		8.07 (3.69)	1–22
SOAS	Survey B (106)	40.1 (5.99)	26–57
Mental		14.8 (3.56)	8–25
Bodily		15.6 (3.01)	6–23
Social		9.60 (1.94)	5–14
IRI	Survey B (106)	92.3 (10.7)	50–116
Perspective		24.3 (5.25)	9–35
Fantasy		23.1 (5.11)	7–35
Concern		24.6 (3.94)	14–33
Distress		20.3 (5.42)	7–31
RSES	Survey C (153)	29.6 (6.53)	12–46
GSES	Survey C (153)	10.3 (4.96)	0–22

*ESSS, Embodied Sense of Self Scale; O-LIFE, The Oxford Schizotypal Personality Scale; SOAS, Sense of Agency Scale; IRI, Interpersonal Reactivity Index; RSES, Rosenberg Self Esteem Scale; GSES, Generalized Self Efficacy Scale.*

*Please see references for the meaning of sub-scores in each existing questionnaire.*

### Confirmation of Reliability (Survey A): Internal Consistency

In order to confirm the internal consistency of the 25-item ESSS, α coefficients were calculated for the total score and each factor score among the 718 university students sample. The results indicate sufficient values for practical usage of the ESSS: α = 0.84 for total, 0.74 for *Ownership*, 0.75 for *Narrative*, and 0.71 for *Agency*.

### Confirmation of Validity (Survey B): Convergent and Divergent Validity

Survey B examined the relationship between the ESSS and other existing scales among 106-member community sample. For the convergent validity, the correlations between the ESSS and O-LIFE (schizotypy) /SOAS (sense of agency) in their total scores were positively significant (*rs* > 0.67) (see **Table 3** for correlations among sub-scores of scales). On the other hand, the correlation between ESSS and IRI (empathy) was also significant (*r* = 0.46), but the effect size was smaller than the above values, indicating the divergent validity. In particular, from the sub-score results (**Table 3**), we see that the ESSS was significantly correlated with two factors of the IRI: Fantasy and Distress. Since these two factors have been suggested to correlate with schizotypal personality (Asai et al., 2011), the correlation between the ESSS and IRI might be a spurious correlation, mediating schizotypy. Indeed, when the schizotypy score (total O-LIFE) was partialled out (i.e., partial correlation), the above-mentioned significant correlation between the ESSS and IRI mostly disappeared, while the correlation between the ESSS and SOAS remained significant (**Table 4**). This indicates that the ESS, the awareness for the self, is independent from empathy, the awareness of others. These results, in summary, suggested the criterion-related (convergent and divergent) validity of the ESSS.

**Table 3:** Pearson’s inter-correlation matrix among scales.

	O-LIFE	Positive	Negative	Disorganized	Impulsive	SOAS	Mental	Bodily	Social	IRI	Perspective	Fantasy	Concern	Distress
ESSS	0.69^∗∗^	0.61^∗∗^	0.04	0.64^∗∗^	0.36^∗∗^	0.81^∗∗^	0.70^∗∗^	0.67^∗∗^	0.18	0.46^∗∗^	0.18	0.33^∗∗^	0.09	0.35^∗∗^
Ownership	0.57^∗∗^	0.61^∗∗^	0.04	0.50^∗∗^	0.19^∗^	0.64^∗∗^	0.54^∗∗^	0.52^∗∗^	0.17	0.32^∗∗^	0.13	0.23^∗∗^	0.06	0.24^∗^
Narrative	0.72^∗∗^	0.60^∗∗^	0.65^∗∗^	0.06	0.49^∗∗^	0.77^∗∗^	0.64^∗∗^	0.65^∗∗^	0.18	0.32^∗∗^	0.13	0.23^∗∗^	0.06	0.24^∗^
Agency	0.45^∗∗^	0.33^∗∗^	–0.02	0.49^∗∗^	0.23^∗^	0.66^∗∗^	0.61^∗∗^	0.53^∗∗^	0.10	0.43^∗∗^	0.18	0.16	0.12	0.43^∗∗^

*^∗∗^p < 0.01, ^∗^p < 0.05.*

*ESSS, Embodied Sense of Self Scale; O-LIFE, The Oxford Schizotypal Personality Scale; SOAS, Sense of Agency Scale; IRI, Interpersonal Reactivity Index.*

**Table 4:** Partial-correlation controlling O-LIFE total score.

	SOAS	Mental	Bodily	Social	IRI	Perspective	Fantasy	Concern	Distress
ESSS	0.65^∗∗^	0.57^∗∗^	0.42^∗∗^	0.05	0.24^∗^	0.13	0.03	0.07	0.23^∗^
Ownership	0.42^∗∗^	0.37^∗∗^	0.25^∗∗^	0.07	0.09	0.07	–0.04	0.02	0.10
Narrative	0.56^∗∗^	0.48^∗∗^	0.38^∗∗^	0.05	0.20^∗^	0.11	0.20^∗^	0.02	0.07
Agency	0.54^∗∗^	0.50^∗∗^	0.36^∗∗^	0.00	0.29^∗^	0.13	–0.05	0.10	0.35^∗∗^

*^∗∗^p < 0.01, ^∗^p < 0.05.*

*ESSS, Embodied Sense of Self Scale; O-LIFE, The Oxford Schizotypal Personality Scale; SOAS, Sense of Agency Scale; IRI, Interpersonal Reactivity Index.*

### Confirmation of Validity (Survey C): In Relation with Similar Concepts

Survey C examined the relationship with similar concepts for the self among the 153-member community sample. The well-accepted concept of self-efficacy means self-evaluation of the ability to perform necessary behaviors according to the situation (Sherer et al., 1982). The concept of self-esteem means self-respect and therefore high evaluation (Rosenberg, 1965). These scores should be modulated by the ESSS since the ESS was developed to comprehend not only the sensory level of self (minimal self) but identity and personality (narrative self) so that self-efficacy and self-esteem should be more related to *Narrative* than *Ownership* or *Agency*. Results indicated that is the case, where the negative correlations were observed between the total ESSS and GSES (self-efficacy)/RSES (self-esteem) (*r* = -0.28 and -0.30, respectively). Since these relationships were not so strong, the ESSS and GSES/RSES are not sharing totally the same concepts but rather an anomalous ESS might entail weaker self-esteem and self-efficacy because these two concepts showed the strongest correlation with *Narrative* (**Table 5**). Narrative self means the temporal extension of minimal self. An anomalous narrative self, that includes identity or personality, would decrease our positive self-evaluation (self-respect or self-esteem), where we recognize ourselves as a less unified and inconsistent representation of the self. These contrasting results indicate factorial validity in terms of factor interpretation.

**Table 5:** Inter-correlation among Embodied Sense of Self, Self-Esteem, and Self-Efficacy.

	RSES	GSES
ESSS	–0.28^∗∗^	–0.30^∗∗^
Ownership	–0.24^∗∗^	–19^∗^
Narrative	–0.36^∗∗^	–0.37^∗∗^
Agency	–0.17^∗^	–0.24^∗^

*^∗∗^p < 0.01, ^∗^p < 0.05.*

*ESSS, Embodied Sense of Self Scale; RSES, Rosenberg Self Esteem Scale; GSES, Generalized Self Efficacy Scale.*

### Confirmation of Reliability (Survey D): Re-test Reliability

Survey D examined the re-test reliability of ESSS among the same 132 participants in Survey C. The interclass correlation coefficients between the two surveys with a month interval showed high enough values for practical use: *r*_ICC_ = 0.82 for total, 0.84 for *Ownership*, 0.81 for *Narrative*, and 0.78 for *Agency*.

### Confirmation of Validity (Survey E): Clinical Validity

**FIGURE 1:**
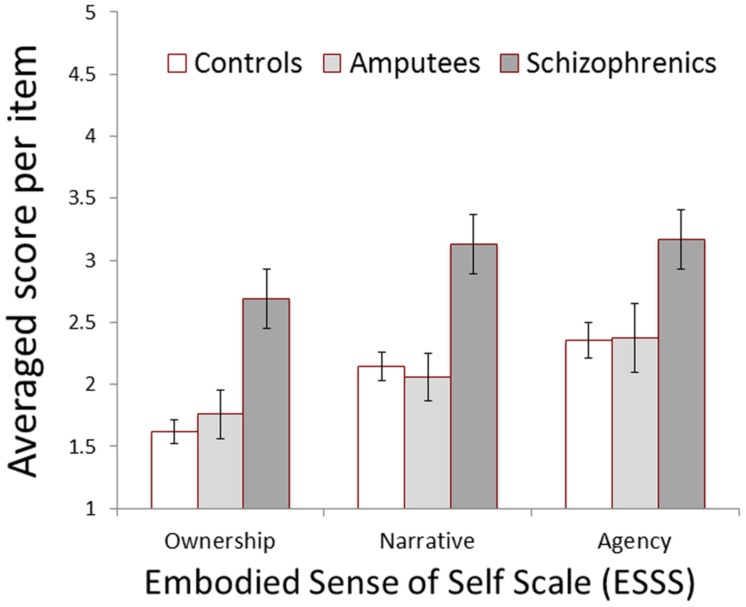
**The difference in Embodied Sense of Self Scale (ESSS) among clinical groups.** Controls = age-matched healthy controls (*N* = 32), Amputees = upper-limb amputees (*N* = 11), Schizophrenics = chronic schizophrenia patients (*N* = 15), and Error bars = ±1SE.

One application of the ESSS is to simply evaluate people with an anomalous ESS and the effect of rehabilitation and intervention in a more objective way. For that purpose, we examined the clinical validity of the ESSS, whether the ESSS could differentiate people with anomalous self-representation like schizophrenia. Fifteen schizophrenic patients, 11 amputees, and 32 healthy controls answered the ESSS. **Figure 1** shows the group difference in the ESSS score. A two-way ANOVA with sub-scores (i.e., factors) as the within-subject independent variable, groups as the between-subject independent variable, and averaged score per item as the dependent variable revealed that the interaction was not significant [*F*(4, 110) = 0.50, *p* > 0.70] but the main effect of groups [*F*(2, 55) = 11.82, *p* = 0.00] as well as the main effect of factors [*F*(2, 4) = 17.23, *p* = 0.00] were significant. Regarding the difference among groups, a post-hoc multiple comparison using Ryan’s method (the most powerful in statistical power among the commonly used multiple comparison methods, Kirk, 1995) revealed significant differences between schizophrenics and amputees/controls (*ps* < 0.05). Regarding the difference among factors, Ryan’s method revealed the difference between *Ownership* and *Narrative*/*Agency* (*ps* < 0.05). Even when a one-way ANOVA with groups as the between-subject independent variable was repeated for each factor, the statistical results were the same: there was a significant difference between schizophrenics and amputees/controls, regardless of factors (*ps* < 0.05). These results indicate that the ESSS selectively predicted anomalous self-representation in people with schizophrenia, suggesting its predictive and clinical validity.

## Discussion

The current study focused on the concept of the ESS, which has attracted researchers in various fields, and revealed its factorial structure. With reference to previous studies, we first collected 120 potential items. Through statistical processes, we finally developed a 25-item ESSS with three factors: *Ownership*, *Narrative*, and *Agency*. These extracted factors match our hypothesis wherein the notions of Gallagher, Ramachandran and Damasio were considered (Ramachandran, 1998; Damasio, 1999; Gallagher, 2000). As a scale for practical use, the ESSS’s validity and reliability were confirmed from multiple perspectives (criterion-related validity, factorial validity, clinical validity, internal consistency, and re-test reliability). In what follows, we discuss each factor in detail.

### Ownership

When it comes to the sense of self, the mechanism for the sense of ownership over our own body has been intensively examined recently. One reason for this is the outstanding and handy phenomenon known as the rubber hand illusion (RHI; Botvinick and Cohen, 1998). The RHI indicates that we would feel an illusory sense of ownership over a fake hand after observing simultaneous brush stroking both on our own hand and a fake hand for a short period, suggesting multi-modal (especially, vision, and somatosensation) integration contributes to our sense of body-ownership (Kilteni et al., 2015). Furthermore, even the out-of-body experience might be caused by electrical stimulation of a specific brain area (i.e., temporal–parietal junction) that is responsible for such multi-modal integration (Blanke, 2004). These studies have suggested that multi-modal integration “constructs” our sense of ownership over our body. Therefore, we can predict that when that integration fails for any reason, that sense for body might also be disturbed. That is the case in schizophrenia. People with schizophrenia or higher schizotypal personality even in the general population have been reported to be more susceptible to RHI (Peled et al., 2000; Asai et al., 2011). This might be because they have a weaker sense of ownership over their own body due to a disturbed multi-modal integration. Indeed in the current study, the *Ownership* score was positively correlated to the schizotypy score, especially to positive symptomatology (**Table 3**). This is consistent with the notion that a positive symptom is a result of the disorder of self-representation (Frith, 2005; Cermolacce et al., 2007) (see also *Embodied Sense of Self* below).

The concept of the sense of ownership has been extended recently. Some might refer to a special term of the sense of “body”-ownership, which means ownership over our own body. However, our body might be just an operational tool for brain, like other external tools, though our body has a hard-wired connection with the brain (“body-as-subject” or “body-as-object”, Sivertsen and Normann, 2015). Operable objects can be attributed to ourselves as our own body (Maravita and Iriki, 2004), in the service of “distributed cognition” for tool use (Baber et al., 2014). Further, we can have an extended sense of ownership over the existence or reality of ourselves. Some people with depersonalization, who have a disorder in integration of perception, motor, memory, and hence identity and who then have no reality for themselves, lose this extended sense of ownership as well as the sense of body-ownership (Putnam, 1997; Sierra and Berrios, 2000). The current study collected items for *Ownership* in line with these discussions. As a result, in addition to our body, the sense of existence or reality as well as our thoughts and tools (i.e., cloths) are included in this factor, suggesting the extended concept of *Ownership*, where ownership over the body is expanding its target to include higher cognition.

There might be some mechanisms for achieving the sense of ownership, depending on its target. As mentioned above, the sense of body-ownership is derived from multi-modal integration (visuo-somatosensary passive integration). In order to expand its target, there should be an additional function, since we cannot get a somatosensation from external objects unless an illusory situation like the RHI is applied. One possible mechanism for extended ownership is controllability of the target (e.g., tool embodiment, Maravita and Iriki, 2004). The controllability is closely related to *Agency* (Haggard and Chambon, 2012, discussed below), where we can learn how to manipulate objects and get a sense of controllability. The sense of agency or controllability would entail the sense of ownership over that object in turn (Asai, 2015a). Since we feel ownership even over external objects, which have no somatosensation, by manipulating them (active integration between motor predication and sensory feedback), this is referred to as the “override” of ownership by agency (Tsakiris et al., 2006). In other words, we feel ownership too when agency is elicited. Next, we will see that *Agency* has a close relationship with *Ownership*.

### Agency

The relationship between agency and (body-) ownership is complicated as mentioned above, since they might have a cross-referred relationship (Tajadura-Jimenez et al., 2012; Asai, 2015a,b; Serino et al., 2015). They are sometimes regarded as a set (e.g., minimal self; Hur et al., 2014), while they can be dissociated conceptually (Gallagher, 2000), phenomenologically (Longo et al., 2008) and in brain activity (Tsakiris et al., 2010). The simple theoretical difference between them is whether our own motor system is involved. The sense of ownership is elicited even when others raise our hand (“the raised hand is my own hand”) while the sense of agency is elicited only when we ourselves raise our hand (“I am raising my hand”). Though agency and ownership are caused simultaneously in voluntary actions (so they are confusing and difficult to distinguish even empirically), *Agency*, unlike *Ownership*, needs our own action and intention that precedes that action. Indeed, in the current study, they were statistically extracted separately as independent factors. When it is difficult to manipulate our own body, agency over body is lost. When it is difficult to manipulate external tools, agency over that tool is lost. In that situation, we might not be aware of the intention for that action and more seriously entail a failure of the desired action (**Table 1**), that people with schizophrenia exhibit (e.g., Walker, 1994).

Agency has been examined in tandem with schizophrenia historically. Though now we can regard schizophrenia as a disorder of the ESS, not solely as one of *Agency*, since some studies have also indicated a disorder in *Ownership* or *Narrative*, the concept of *Agency* started with schizophrenia. Many studies have examined their reported agency during motor tasks including hand action, speech production, or cursor manipulation (e.g., Daprati et al., 1997; Franck et al., 2001; Johns et al., 2010). Though the results are not always consistent, a meta-analytic study concluded that patients with schizophrenia have a disturbed agency (“self-recognition” in action, Waters et al., 2012). In the current study, *Agency* was positively correlated with schizotypal scores. Furthermore, even when the schizotypy score was controlled, *Agency* was still correlated with the agency scale (SOAS). This suggests that anomalous agency is not a schizotypal experience itself. Rather, we have a function of agency, independent of schizotypal experiences, and an expression of its disturbance would be schizotypal symptomatology.

The mechanism for agency is thought to be related to our motor control system. Since we have learned the controllability of our own body from birth, we have achieved the input-output coordination where we can predict how the body moves when the motor command is given. The sense of agency is the feeling that evaluates how successfully such an intentional action is executed. Therefore, not only for our body but for external tools whose controllability we can learn, we feel agency. Also as a result in that situation, we feel ownership over the tools as mentioned above. In this way, agency and ownership have a complicated but necessary interaction so that they construct a sensory level of self-representation (i.e., minimal self). Further studies should still examine how they interact while sometimes overriding each other, and how they construct the minimal self during a short temporal scale (Imaizumi and Asai, 2015; White, 2015).

### Narrative

Narrative self, with its being achieved over a long temporal scale (i.e., years), has received less attention than minimal self (but White, 2015). Though Gallagher (2000) suggested a further necessity to examine this aspect of self, the sense of continuity and uniformity would be potential keywords for this. Specifically, autobiographical source memory and prospective decision making for action in terms of self-representation have already been examined recently. Through these studies, we should reveal in the future how the sensory self (i.e., minimal self) at a specific moment, for example, “I am doing this action”, would be retained in our memory, “I did that action”, and would determine the next action, “I will do that action”.

Theoretically, the narrative self is a temporal extension (i.e., continuity and uniformity) of the minimal self, indicating that *Narrative* might be a crystallized self-representation including *Ownership* and *Agency*. In this sense, the narrative self is also embodied, where we refer to ourselves as identical entity through memorized experiences based on body and action. Though schizophrenic symptomatology has been suggested to be linked with *Ownership* (e.g., RHI), *Agency* (e.g., action recognition) and *Narrative* (e.g., source memory) (see also Section “Embodied Sense of Self” below), schizotypy scores showed stronger correlation with *Narrative* than *Ownership*/*Agency* in the current study (**Table 3**). *Narrative* also showed the strongest correlation with self-evaluation (meta self-representation) like self-esteem and self-efficacy (**Table 5**). In line with the theory, these results might indicate that narrative self, including continuity and uniformity, is a higher or integrated function for self-representation based on the minimal self.

In that case, the question here is how the minimal self develops the narrative self, with achieving temporal extension (Damasio, 1999; Gallagher, 2000). A possible mechanism is “self-labeling” of sensory information. The minimal self basically depends on online sensory information, where each sensation is distinguished between its being self-related or not. For example, the brain specifically responds to this self-related information, such as our own name, face, or objects like a favorite cup (Legrand and Ruby, 2009; Miyakoshi et al., 2010) where this visual or auditory information is given a self-label, that is derived from the sense of ownership. On the other hand, we can’t tickle ourselves (Blakemore et al., 1998) because self-originated sensory input (i.e., sensory feedback) is suppressed (Miall and Wolpert, 1996). This helps us to save our attention resource or prevent desensitization (Poulet and Hedwig, 2002). We can say that that sensory feedback is given a self-label that is derived from the sense of agency. The narrative self might utilize self-labels on sensory information, not the sensory information itself (c.f., Miyakoshi et al., 2008; Quiroga et al., 2008; Habermas and Kober, 2015). For example, when we can “hit” our own action in a recognition task of source memory (i.e., when we correctly judge “this is what I did”), we feel more agency for that action in the learning phase (i.e., during action execution). This indicates an important contribution of the online minimal self to the narrative self, where self-labels are used. Though the current study extracted three (semi-) independent factors—*Ownership*, *Narrative* and *Agency*—in terms of the *Embodied Sense of Self*, they should interact with each other (cross-reference, compensation, overriding, etc.), as we can see among previous studies. Further study should examine the relationship among those factors in order to reveal the hierarchic structure of unified self-representation.

### Embodied Sense of Self

The current study suggested that the ESS consists of the above-mentioned three factors, focusing on the everyday experiences that an anomalous ESS would entail. Survey E indicates that people with schizophrenia have more such experiences on a daily basis, aside from their acute symptoms themselves, regardless of factors (**Figure 1**). This finding is consistent with previous studies where an anomalous ownership, agency, or narrative self have been reported for schizophrenia empirically (e.g., Daprati et al., 1997; Peled et al., 2000; Wang et al., 2011; Shakeel and Docherty, 2012). Though schizophrenic symptomatology, especially positive symptoms, had initially been regarded as a disorder in agency, now we should regard it as a disorder of the ESS, where each factor interacts in a complicated manner but finally constructs a unified representation of the self. That factorial relationship, however, is still unclear. Untying that complexity would also help us understand the psychopathology of schizophrenia and other mental disorders in the future. A larger sample would be necessary for that purpose. This simple and easy evaluation tool will be especially useful for clinical studies.

The scientific interest in the self is not new. Researchers have been exploring the origin of the self for decades, but that question still remains the ultimate mystery. The new challenge for this old but new question is the embodiment approach (Niedenthal et al., 2005). As brain imaging techniques progress, we might have gotten the idea in the “brain era” that processing for everything, including the self, is embedded within our brain (c.f., “brain in a vat”, Hilary, 1982). The embodiment approach (e.g., Damasio, 1999, 2001; Bargh et al., 2001; Pfeifer et al., 2007; Taylor et al., 2009; Blanke et al., 2015; Caspar et al., 2015), however, considers that the brain is just for the body and action. This view has attracted many researchers because a measurable index is the essential factor for the self; that is, body and action themselves are the self, not just a proxy. When we tackle the self-specific brain activity, methodological limitations (i.e., s/n ratio, spatial resolution, etc.) are also problematic (Legrand and Ruby, 2009). In this sense, we first have to explore the structure of the subjectivity of the self in terms of embodiment so that we would know how each factor for the self is represented in our brain in the future. We still don’t know yet whether this approach takes us higher, but we hope this new tool for measuring the ESS (Supplemental Material S2) will contribute to finally coming to a conclusion.

## Author Contributions

TA, NK designed the study. TA, NK, SI, SK, and SK performed the surveys. TA analyzed the results and wrote the manuscript. All authors approved the final version of the manuscript.

## Conflict of Interest Statement

The authors declare that the research was conducted in the absence of any commercial or financial relationships that could be construed as a potential conflict of interest.
