# Supplementary material for: Development of Embodied Sense of Self Scale (ESSS): Exploring Everyday Experiences Induced by Anomalous Self-Representation
Source: Front Psychol. 2016 Jul 5;7:1005. doi: 10.3389/fpsyg.2016.01005 (PMC4932106; doi:10.3389/fpsyg.2016.01005)
Supplement: Supplementary file 1 [file Table_1.PDF]

1

Please indicate the extent to which the following statements generally apply to you by circling the corresponding number (1-5) next to the statement.

For example: I tire easily. 1 2 (3) 4 5

		Strongly disagree	Disagree somewhat	Neither disagree nor agree	Agree somewhat	Strongly agree
1	Sometimes I forget what I was going to say.	1	2	3	4	5
2	Sometimes I just feel like moving unconsciously.	1	2	3	4	5
3	I tend to worry that I may have forgotten to lock the door, turn off the air conditioner, etc.	1	2	3	4	5
4	Sometimes I feel it is impossible to know what others are thinking.	1	2	3	4	5
5	I first become aware of my own body when I first touch myself.	1	2	3	4	5
6	Things I use everyday such as cups and pens feel just like they are a part of me.	1	2	3	4	5
7	Something may attach itself to me without me realizing it.	1	2	3	4	5
8	When my mood changes, I cannot remember what I was thinking before.	1	2	3	4	5
9	Sometimes what I think are my own ideas are actually someone else's ideas.	1	2	3	4	5
10	I feel like my personality is unique and distinct from others.	1	2	3	4	5
11	I can't tell from taste or smell whether food is good or bad, pleasant or unpleasant.	1	2	3	4	5
12	Sometimes I feel stiff for a while.	1	2	3	4	5
13	Sometimes I tend not to notice incoming calls when my cell phone is set to vibration mode.	1	2	3	4	5
14	Close physical approach of another person make me feel uncomfortable.	1	2	3	4	5
15	It annoys me when people misuse things even though it's stuff I threw away or no longer need.	1	2	3	4	5
16	It is difficult to grope around for something without being able to see it.	1	2	3	4	5
17	I have been told that my voice is too loud.	1	2	3	4	5
18	I lose things without even being aware they are lost.	1	2	3	4	5
19	Sometimes it seems as if my own name does not belong to me.	1	2	3	4	5
20	Sometimes it seems as if I am hearing my voice from a distance.	1	2	3	4	5
21	I have a high pain threshold even when I hurt myself by banging into things.	1	2	3	4	5
22	Sometimes things around me seem unusually small (or large).	1	2	3	4	5
23	Sometimes it seems like some part of my body is dead.	1	2	3	4	5
24	I don't always recognize my own face as me.	1	2	3	4	5
25	I sometimes feel as if I have been possessed by something.	1	2	3	4	5
26	No matter how hard I concentrate, unrelated thoughts intrude upon my thinking.	1	2	3	4	5
27	I sometimes feel as if the size of my hands or feet has slightly changed.	1	2	3	4	5
28	Sometimes when I look in the mirror my face appears different than it usually does.	1	2	3	4	5
29	I sometimes feel as if my body doesn't actually exist.	1	2	3	4	5
30	When acting, I get totally into the character I am playing.	1	2	3	4	5
31	I easily get motion sickness.	1	2	3	4	5
32	I feel other peoples' pain even though they are the ones who are physically hurt.	1	2	3	4	5
33	I sometime confuse what I actually said with what I intended to say.	1	2	3	4	5
34	When I see someone getting tapped on the shoulder, it feels like it is me being tapped.	1	2	3	4	5
35	During winter, my hands, feet and ears get cold very easily.	1	2	3	4	5
36	Sometimes it seems as if my body is moving in ways I do not intend.	1	2	3	4	5
37	I have a tendency to bump into things, knock things over, and drop things.	1	2	3	4	5
38	I think I have a strong identity.	1	2	3	4	5
39	I don't feel pain when I see images of people getting injured, operated on, or injected.	1	2	3	4	5
40	I sometime recall things that make me smile to myself.	1	2	3	4	5

2

Please indicate the extent to which the following statements generally apply to you by circling the corresponding number (1-5) next to the statement.

For example: I tire easily. 1 2 (3) 4 5

1	Sometimes words pop out of my mouth before I think what I am going to say.	1	2	3	4	5
2	Sometimes my body feels extremely heavy.	1	2	3	4	5
3	Thoughts that come to mind seem to be those of someone else's.	1	2	3	4	5
4	In the online world of the Internet I behave like a totally different character.	1	2	3	4	5
5	When a stranger sits down right next to me, I feel kind of creepy on that side.	1	2	3	4	5
6	I sometimes dream of escaping from my body.	1	2	3	4	5
7	It seems like the person I was in the past and the person I am today are completely different.	1	2	3	4	5
8	When I close my eyes and let my mind drift, I feel like I am floating.	1	2	3	4	5
9	I do not remember many events from childhood.	1	2	3	4	5
10	I have misperceived things that I should have actually witnessed.	1	2	3	4	5
11	Sometimes I feel that I no longer know my own personality.	1	2	3	4	5
12	Sometimes I mumble to myself as I mull things over.	1	2	3	4	5
13	The distinction between me and other things is not always clear.	1	2	3	4	5
14	Sometimes I can't seem to manipulate the mouse right that controls the cursor.	1	2	3	4	5
15	Sometimes it feels like there is someone behind me.	1	2	3	4	5
16	I can immediately recall what I had for dinner last night.	1	2	3	4	5
17	Imagined events sometimes feel just as real as actual events.	1	2	3	4	5
18	Sometimes it feels like my body is jerky like a robot.	1	2	3	4	5
19	It's fun to interact with anonymous strangers at online gaming sites.	1	2	3	4	5
20	I have misheard static noise on occasion thinking it's people talking.	1	2	3	4	5
21	It feels like I can manipulate a baseball bat or tennis racket just like they are extensions of my arm.	1	2	3	4	5
22	I sometimes bump into things or people when I am out walking.	1	2	3	4	5
23	Sometimes my body goes numb for no particular reason.	1	2	3	4	5
24	When I drive, I tend to veer either to the right or left side of the road.	1	2	3	4	5
25	Real events sometimes seem like dreams even though they actually occurred.	1	2	3	4	5
26	When I am doing something, it seems like I am observing myself from a distance.	1	2	3	4	5
27	I feel the character's pain when I see someone get hurt in a movie or in a drama.	1	2	3	4	5
28	Sometimes the clothes I am wearing feel heavy.	1	2	3	4	5
29	It sometimes seems like the cellphone in my pocket or bag is vibrating.	1	2	3	4	5
30	Sometimes it seems like my soul has become separated or detached from my body.	1	2	3	4	5
31	Sometimes inanimate objects and shadows appear to be human beings.	1	2	3	4	5
32	I am strangely bothered by the way clothing rubs against my skin.	1	2	3	4	5
33	I sometimes sense that events covered in the news are caused by me.	1	2	3	4	5
34	When I am really tired, I get motion sickness when I am walking.	1	2	3	4	5
35	I sometimes go for long periods without feeling hungry or thirsty.	1	2	3	4	5
36	When a song gets stuck in my head, it is really hard to turn it off.	1	2	3	4	5
37	Anonymous communication on the Internet makes me comfortable.	1	2	3	4	5
38	Sometimes I sense that my body is very light.	1	2	3	4	5
39	Sometimes I feel like I am in a dream state where I hallucinate and have weird experiences.	1	2	3	4	5
40	I feel a bit embarrassed when someone points me out in a photograph.	1	2	3	4	5

3

Please indicate the extent to which the following statements generally apply to you by circling the corresponding number (1-5) next to the statement.

For example: I tire easily. 1 2 (3) 4 5

1	Sometimes it feels like my body is being manipulated by someone.	1 2 3 4 5
2	It seems like my childhood days were not that long ago.	1 2 3 4 5
3	It seems like sensory feeling in my body or part of my body has gotten weaker.	1 2 3 4 5
4	I think I am more ticklish than other people.	1 2 3 4 5
5	I tend to drop things when I carry things around.	1 2 3 4 5
6	I have a weak tactile sense, so I don't feel myself touching things.	1 2 3 4 5
7	Without any intent on my part, my hand just grabs things with a mind of its own.	1 2 3 4 5
8	Sometimes I sense hearing someone call my name in a crowd, and I turn around to look.	1 2 3 4 5
9	Sometimes I sense my cell phone ringing even though it's not.	1 2 3 4 5
10	Sometimes I do things without thinking.	1 2 3 4 5
11	I can pick up objects and press switches without looking.	1 2 3 4 5
12	Sometimes I feel as if there is a different personality inside me.	1 2 3 4 5
13	I can imagine what my future looks like.	1 2 3 4 5
14	It is easy for me to get totally absorbed in alter ego characters such as Internet avatars.	1 2 3 4 5
15	Looking at my childhood photos, they don't seem to be me.	1 2 3 4 5
16	When out walking, I rarely notice my reflection in mirrors or shop windows.	1 2 3 4 5
17	I can tell my whole life history from birth right up to the present.	1 2 3 4 5
18	Sometimes my existence seems to lack a sense of reality.	1 2 3 4 5
19	I am ticklish even when I tickle myself.	1 2 3 4 5
20	I completely identify with characters in games and novels.	1 2 3 4 5
21	Sometimes, it seems like it is not me who is experiencing a sensation.	1 2 3 4 5
22	I have a hard time parting with possessions, even trivial things.	1 2 3 4 5
23	Sometimes when I am mulling something over, I carry on a dialog in my head.	1 2 3 4 5
24	My personality changes depending on the setting and the situation.	1 2 3 4 5
25	When I watch weightlifters or other shows of strength, I feel infused with strength.	1 2 3 4 5
26	I feel like sometimes people misunderstand my personality.	1 2 3 4 5
27	Sometimes I confuse things in my head with reality.	1 2 3 4 5
28	I don't feel I am very good at parking the car in the garage.	1 2 3 4 5
29	Sometimes I become aware that my body has become chilled.	1 2 3 4 5
30	I sometimes sense that my face is changing.	1 2 3 4 5
31	Sometimes when I get totally engrossed in thought, I hear voices and sounds.	1 2 3 4 5
32	When I shut my eyes and concentrate on my body, it seems that anywhere one part is weak.	1 2 3 4 5
33	I sometimes confuse stories I have heard from others and my out experiences.	1 2 3 4 5
34	I cannot remember what I did during that period because my memory was fuzzy.	1 2 3 4 5
35	I sometimes feel that the shape of my body (or part of my body) is changing.	1 2 3 4 5
36	It sometimes seems as if part of my body—head, arm, leg—is not really part of me.	1 2 3 4 5
37	I sometimes feel that casual conversation and sounds made by strangers is about me.	1 2 3 4 5
38	When I am in a festive mood, I seldom recall sad events.	1 2 3 4 5
39	I sometimes think that events in the world are the result of my own deeds.	1 2 3 4 5
40	I sometimes get dizzy for no apparent reason.	1 2 3 4 5
