# Supplementary material for: Development of Embodied Sense of Self Scale (ESSS): Exploring Everyday Experiences Induced by Anomalous Self-Representation
Source: Front Psychol. 2016 Jul 5;7:1005. doi: 10.3389/fpsyg.2016.01005 (PMC4932106; doi:10.3389/fpsyg.2016.01005)
Supplement: Supplementary file 2 [file Table_2.PDF]

(Japanese version)

1

次の項目に対して，普段のあなた自身にどの程度当てはまるかを，
1～5 に○をつけて回答してください。

例：　すぐに疲れてしまう　　1　2　(3)　4　5

			当てはまらない	あまり当てはまらない	どちらとも言えない	少し当てはまる	当てはまる
1	n	自分の性格が，自分でもよく分からなくなることがある	1	2	3	4	5
2	o	街を歩いていて，鏡やショーウインドウに映った自分に気づかないことがある	1	2	3	4	5
3	a	物を見ずに手探りで探すのは難しいと感じることがある	1	2	3	4	5
4	o	着ている洋服が重いような気がすることがある	1	2	3	4	5
5	a	知らないうちに，落し物をしていることがある	1	2	3	4	5
6	n	どんなに集中しようとしても，関連のない考えが思考に入ってくることがある	1	2	3	4	5
7	a	物を運んでいるときに，落としてしまうことがある	1	2	3	4	5
8	o	頭の中の考えが，自分のものではないように感じることがある	1	2	3	4	5
9	n	昔の自分と今の自分は，まったくの別人であるように感じることがある	1	2	3	4	5
10	a	自分の身体に，知らない間に何かがくっついていても気がつかないことがある	1	2	3	4	5
11	o	自分の身体がロボットであるかのように，ぎこちなく感じることがある	1	2	3	4	5
12	n	自分の性格は，その場面や状況に応じて変わると感じる	1	2	3	4	5
13	o	自分が何かしている時，離れているところから自分を眺めているように感じることがある	1	2	3	4	5
14	a	何を言おうとしていたのか忘れてしまうことがある	1	2	3	4	5
15	o	洋服と自分の皮膚が擦れるのが妙に気になることがある	1	2	3	4	5
16	n	音楽が頭の中で聞こえていて，自分ではなかなか消しにくいことがある	1	2	3	4	5
17	o	自分の体がとても軽く感じられることがある	1	2	3	4	5
18	a	他の人よりも，くすぐったがりだと思う	1	2	3	4	5
19	n	思い出し笑いをすることがある	1	2	3	4	5
20	a	声が大きいと言われることがある	1	2	3	4	5
21	o	自分の存在に現実感がない時がある	1	2	3	4	5
22	n	人から言われる自分の性格は何か間違っているように感じる	1	2	3	4	5
23	a	歩いていて物や人にぶつかってしまうことがある	1	2	3	4	5
24	o	気づいたら，自分の体が冷たくなってしまっていることがある	1	2	3	4	5
25	n	その時何をしていたか，記憶があいまいな時期がある	1	2	3	4	5

＊注
・2 列目は因子名：a(agency)＝制御感 8 項目, o(ownership)＝所有感 9 項目, n(narrative)＝物語自己 8 項目
・逆転項目なし。単純加算して，得点の高いほど，その因子の異常，不安定さが高くなる
・因子名（a, o, n）および注は，印刷・実施時には削除してください。

(English version)

1

Please indicate the extent to which the following statements generally apply to you by circling the corresponding number (1-5) next to the statement.

For example: I tire easily.   1   2   (3)   4   5

			Strongly disagree	Disagree somewhat	Neither disagree nor agree	Agree somewhat	Strongly agree
1	n	Sometimes I feel that I no longer know my own personality.	1	2	3	4	5
2	o	When out walking, I rarely notice my reflection in mirrors or shop windows.	1	2	3	4	5
3	a	It is difficult to grope around for something without being able to see it.	1	2	3	4	5
4	o	Sometimes the clothes I am wearing feel heavy.	1	2	3	4	5
5	a	I lose things without even being aware they are lost.	1	2	3	4	5
6	n	No matter how hard I concentrate, unrelated thoughts intrude upon my thinking.	1	2	3	4	5
7	a	I tend to drop things when I carry things around.	1	2	3	4	5
8	o	Thoughts that come to mind seem to be someone else's.	1	2	3	4	5
9	n	It seems like the person I was in the past and the person I am today are completely different.	1	2	3	4	5
10	a	Something may attach itself to me without me realizing it.	1	2	3	4	5
11	o	Sometimes it feels like my body is jerky like a robot.	1	2	3	4	5
12	n	My personality changes depending on the setting and the situation.	1	2	3	4	5
13	o	When I am doing something, it seems like I am observing myself from a distance.	1	2	3	4	5
14	a	Sometimes I forget what I was going to say.	1	2	3	4	5
15	o	I am strangely bothered by the way clothing rubs against my skin.	1	2	3	4	5
16	n	When a song gets stuck in my head, it is really hard to turn it off.	1	2	3	4	5
17	o	Sometimes I sense that my body is very light.	1	2	3	4	5
18	a	I think I am more ticklish than other people.	1	2	3	4	5
19	n	I sometime recall things that make me smile to myself.	1	2	3	4	5
20	a	I have been told that my voice is too loud.	1	2	3	4	5
21	o	Sometimes my existence seems to lack a sense of reality.	1	2	3	4	5
22	n	I feel like sometimes people misunderstand my personality.	1	2	3	4	5
23	a	I sometimes bump into things or people when I am out walking.	1	2	3	4	5
24	o	Sometimes I become aware that my body has become chilled.	1	2	3	4	5
25	n	I cannot remember what I did during that period because my memory was fuzzy.	1	2	3	4	5

*note:

- The second column means factor names: *a* (agency) 8 items, *o* (ownership) 9 items, and *n* (narrative) 8 items.
- No reversed-items, so that higher score for each factor means more anomalous trait of it.
- Factor names (the second column) and this note must be removed before being printed for conducting surveys.
